# Supplementary material for: Characterisation of nonlinear receptive fields of visual neurons by convolutional neural network
Source: Sci Rep. 2019 Mar 7;9:3791. doi: 10.1038/s41598-019-40535-4 (PMC6405885; doi:10.1038/s41598-019-40535-4)
Supplement: Supplementary file 1 — Supplementary Information [file 41598_2019_40535_MOESM1_ESM.pdf]

Supplementary Information of "Characterisation of nonlinear receptive fields of visual neurons by convolutional neural network"

Jumpei Ukita, Takashi Yoshida, and Kenichi Ohki

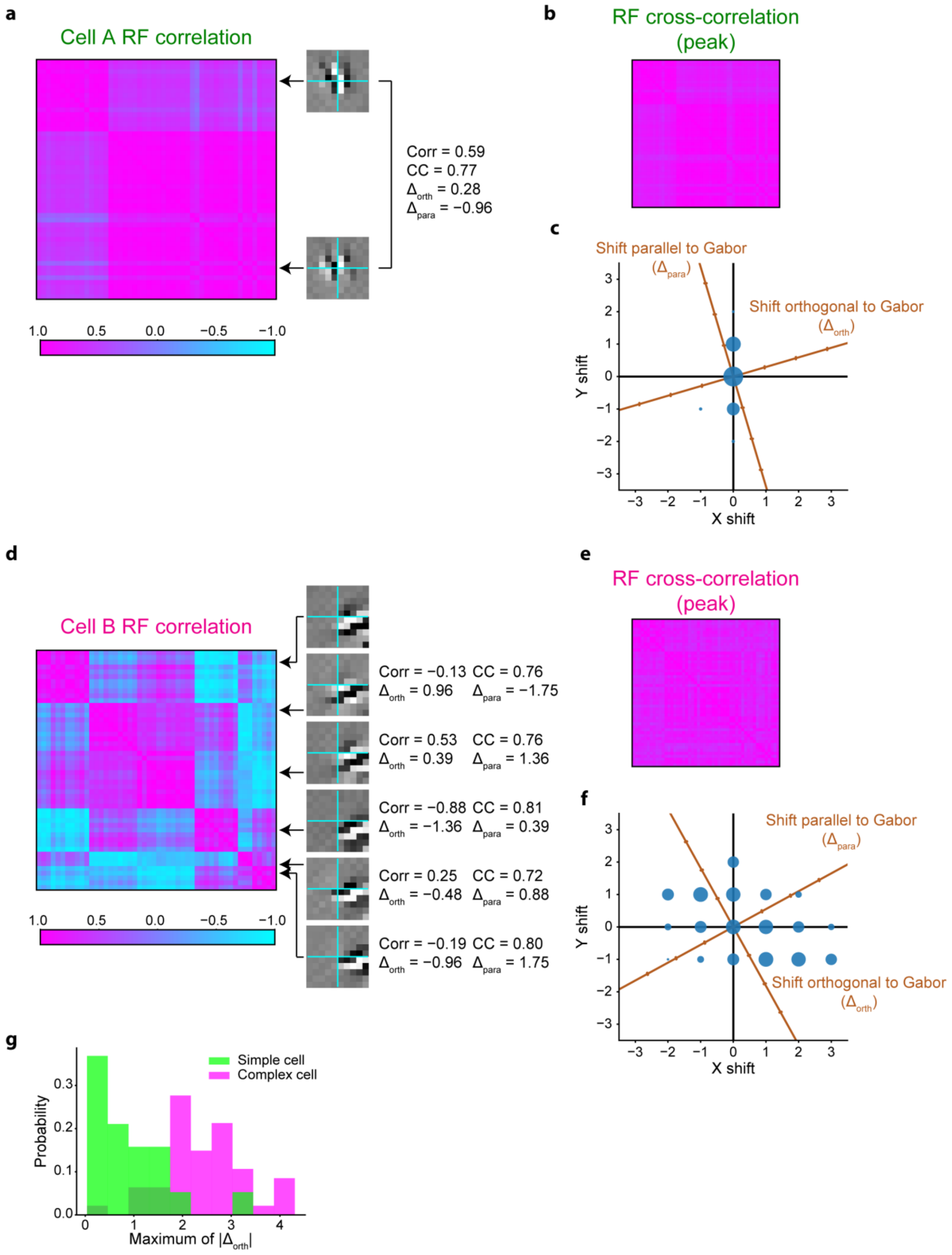

Fig S1. Summary of the estimated RFs for the simulated simple cells and complex cells.

(a, d) Left: correlation matrix of the iteratively visualised RF images of representative simulated simple

cell A (**a**) and complex cell B (**d**). Each row and column correspond to one of the 100 RF images, sorted so that clusters can be clearly visualised. Right: representative RF images. Their correlation, cross-correlation, shift orthogonal to the Gabor orientation, and shift parallel to the Gabor orientation are also shown. For cell B, these metrics were calculated with reference to the top RF image. **(b, e)** Peak cross-correlation matrix of the RF images of each cell. The ordering of RF images and the colour bar are the same as (**a**) or (**e**). **(c, f)** Scatter plot showing the distances with which each pair of RF images were shifted. The size of each scatter corresponds to the probability. The x- and y-axis correspond to the vertical and horizontal axis of the images, respectively. **(g)** Population distribution of the maximum shift distance orthogonal to the Gabor orientation.

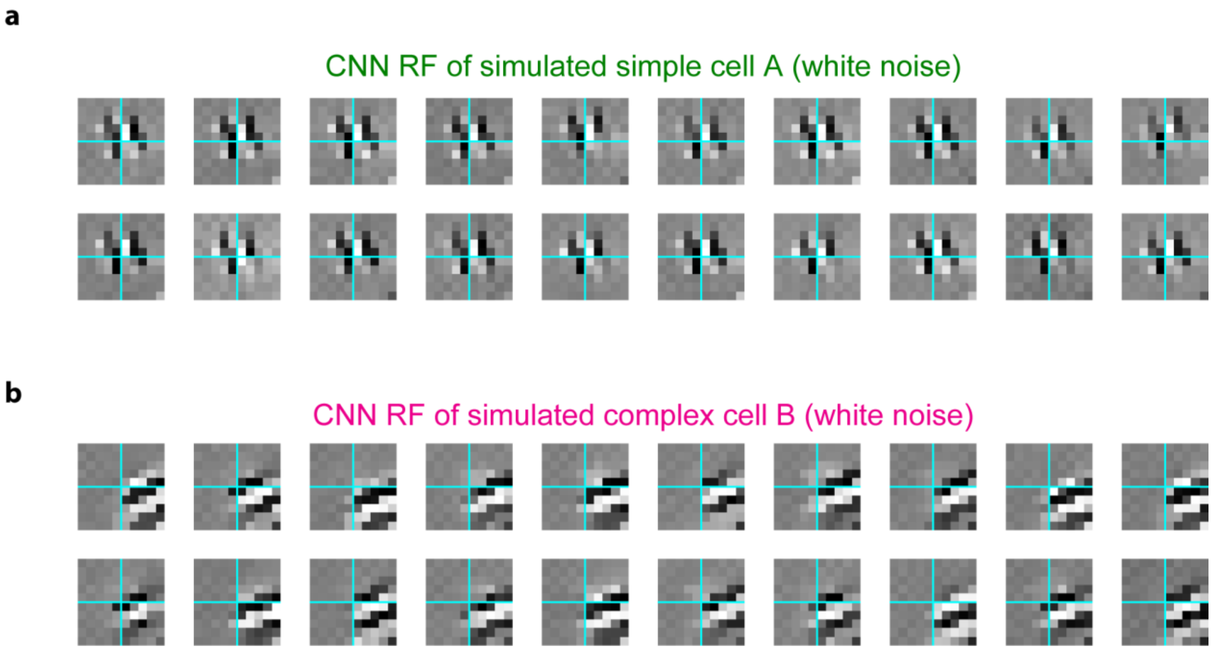

Fig S2. RFs estimated using white noise as the stimuli.

Results of iterative CNN RF estimations for representative cell A (**a**) and cell B (**b**) using a simulated dataset that consists of the white noise images and the corresponding responses. Only 20 out of the 100 generated RF images are shown in this figure.

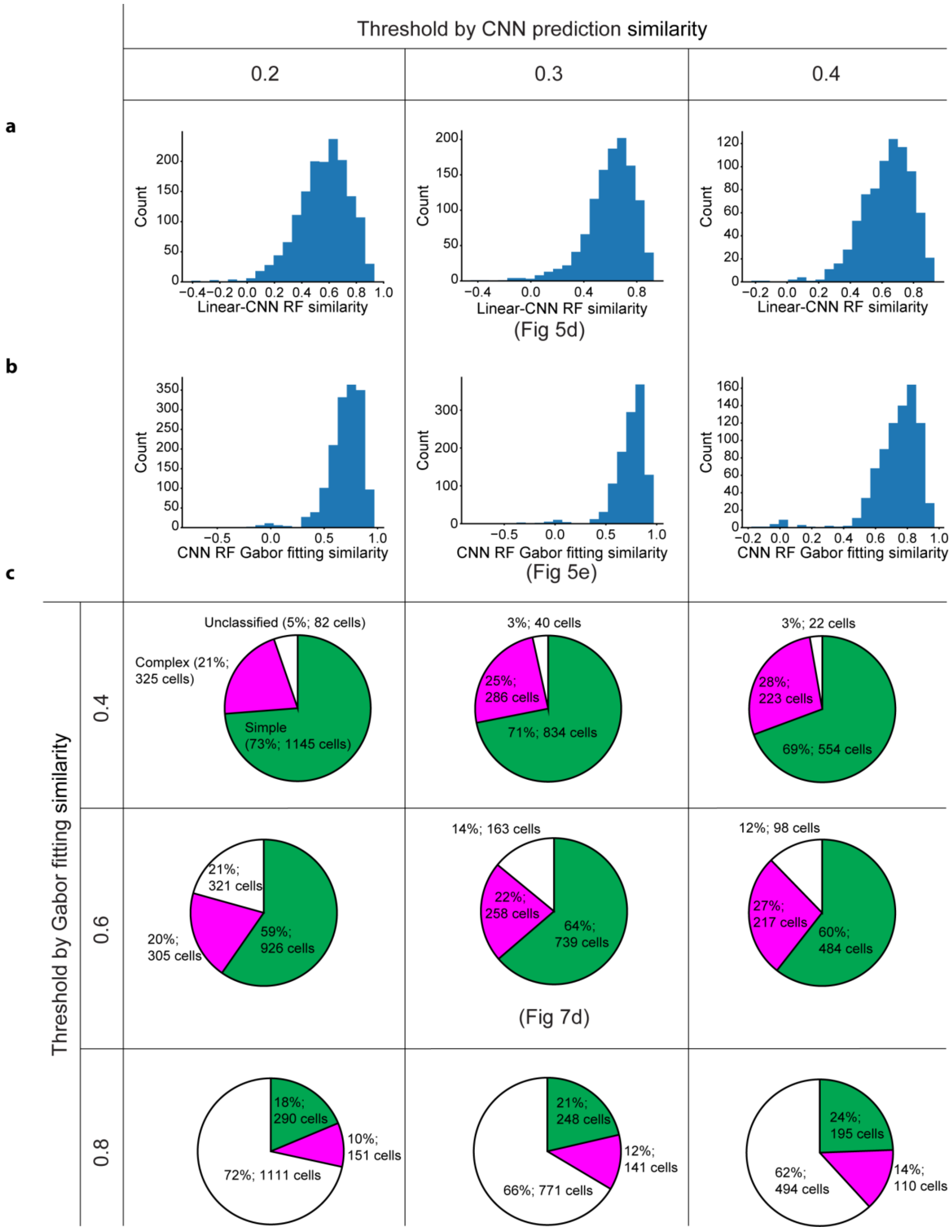

Fig S3. On the dependence of the results on the choice of the thresholds.

(a, b) Similarity between linear RFs and CNN RFs (**a**) and Gabor fitting similarity of CNN RFs (**b**) were

computed using different thresholds for the cell selection. Left: neurons with a CNN prediction similarity > 0.2 were analysed. Middle: neurons with a CNN prediction similarity > 0.3 were analysed. Right: neurons with a CNN prediction similarity > 0.4 were analysed. **(c)** The proportion of classified cells, simple cells, and complex-like cells calculated with different thresholds for the cell selection.

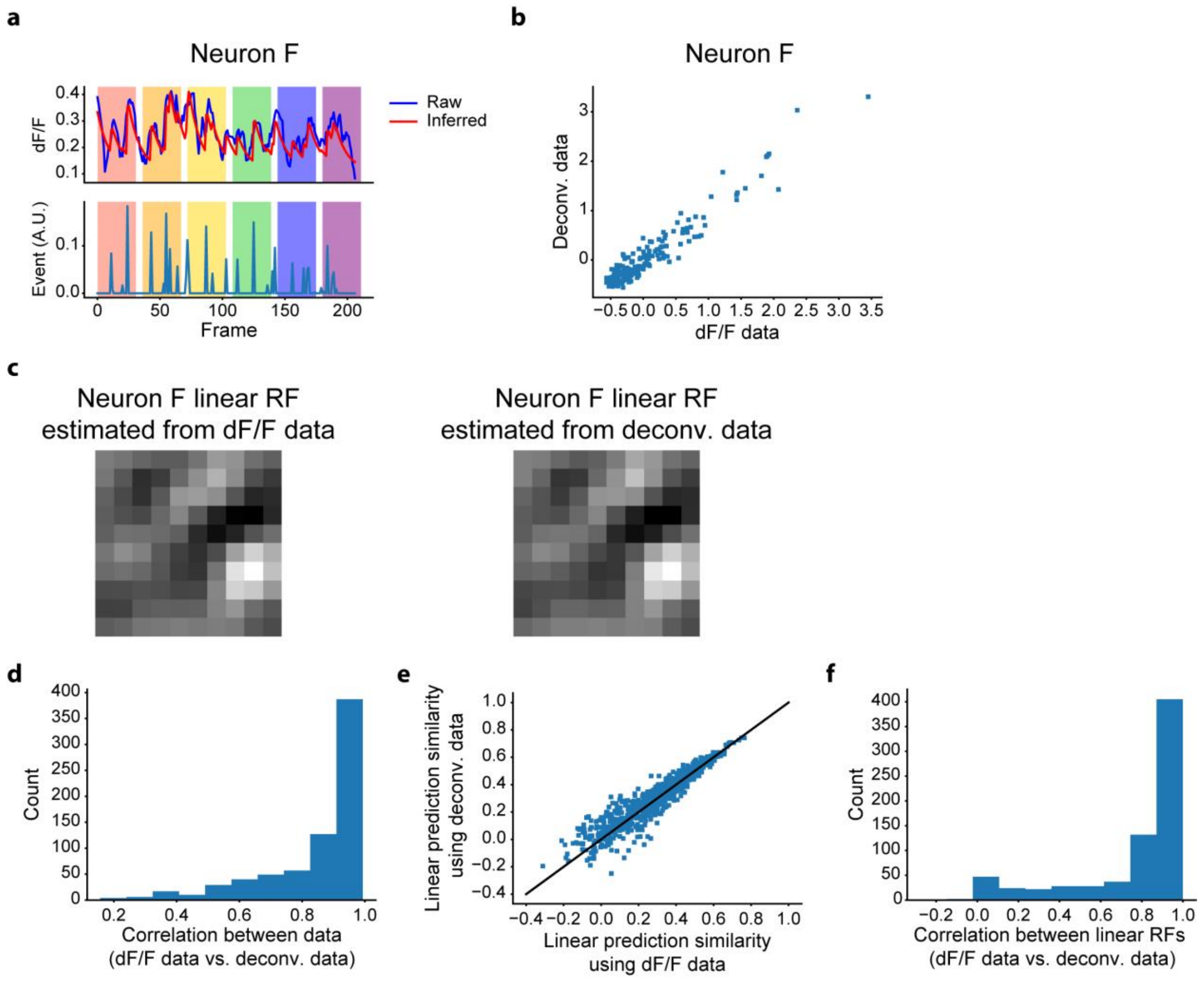

Fig S4. Comparison of linear models using deconvolved data and dF/F data.

(a) Representative calcium trace (top blue), estimated spike events (bottom), and the trace inferred from the estimated events (top red). Different shade colour indicates a different stimulus presented to the animal. **(b)** Relationship of the responses between the dF/F data and deconvolved data for the representative neuron F. **(c)** Linearly estimated RFs using dF/F data (left) and deconvolved data (right) of the representative neuron F. **(d)** Population distribution of the correlation between the dF/F data and the deconvolved data. **(e)** Population scatter plot of the linear prediction similarities using each data. **(f)** Population distribution of the correlation between the linear RFs estimated using each data.

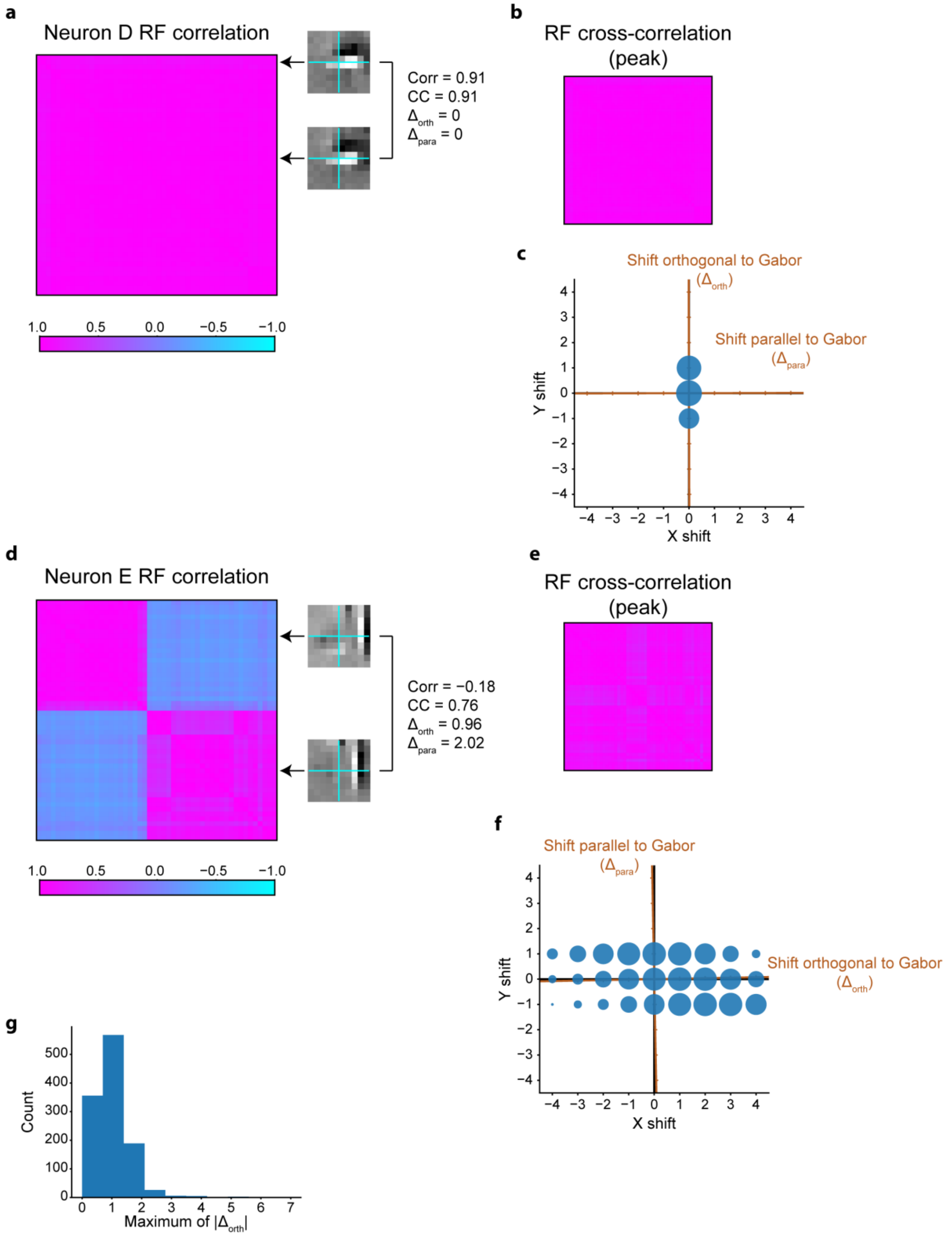

Fig S5. Summary of the estimated RFs for the V1 neurons.

(a, d) Left: correlation matrix of the iteratively visualised RF images of representative neuron D (**a**) and E

(**d**). Each row and column correspond to one of the 100 RF images, sorted so that clusters can be clearly visualised. Right: representative RF images. Their correlation, cross-correlation, shift orthogonal to the Gabor orientation, and shift parallel to the Gabor orientation are also shown. **(b, e)** Peak cross-correlation matrix of the RF images of each neuron. The ordering of RF images and the colour bar are the same as (**a**) or (**e**). **(c, f)** Scatter plot showing the distances with which each pair of RF images were shifted. The size of each scatter corresponds to the probability. The x- and y-axis correspond to the vertical and horizontal axis of the images, respectively. **(g)** Distribution of the maximum shift distance orthogonal to the Gabor orientation.
